# A qualitative view of the HIV epidemic in coastal Ecuador

**DOI:** 10.7717/peerj.2726

**Published:** 2016-11-22

**Authors:** Adam L. Beckman, Magdalena M. Wilson, Vishaal Prabhu, Nicola Soekoe, Humberto Mata, Lauretta E. Grau

**Affiliations:** 1 Yale College, New Haven, CT, United States; 2 Department of Social and Behavioral Sciences, Yale School of Public Health, New Haven, CT, United States; 3 Department of Ethics, Politics and Economics, Yale University, New Haven, CT, United States; 4 Fundación VIHDA, Guayaquil, Ecuador; 5 Center for Interdisciplinary Research on AIDS and the Department of Epidemiology of Microbial Disease, Yale School of Public Health, New Haven, CT, United States

**Keywords:** HIV/AIDS, HIV risk perception, HIV risk behavior, HIV attitudes, Ecuador, Global health, Public health, Latin America, Risk behavior, Qualitative

## Abstract

In 2013 approximately 37,000 people were living with HIV in Ecuador (prevalence 0.4%), representing a generalized epidemic where most new infections arise from sexual interactions in the general population. Studies that examine attitudes towards people living with HIV (PLWH), individual risk perception of acquiring HIV amongst Ecuadorians, and the ways in which levels of risk perception may affect risk behaviors are lacking. This qualitative study aimed to fill this gap in the literature by investigating these issues in the rural, coastal community of Manglaralto, Ecuador, which has among the highest incidence of HIV in Ecuador. We conducted interviews with 15 patients at Manglaralto Hospital. Analysis of interview transcripts revealed widespread negative attitudes towards PLWH, prevalent risk behaviors such as multiple sex partners and lack of condom use, and low individual risk-perception of contracting HIV. These findings underscore the need for increased efforts to prevent further growth of the HIV epidemic in Ecuador.

## Introduction

Available data show that the HIV epidemic in Ecuador primarily affects heterosexual people living in the coastal regions of the country. In 2013 there were approximately 37,000 people living with HIV (PLWH) in Ecuador, representing a prevalence of about 0.4% of adults ages 15–49 (UNAIDS, 2013). The majority of cases of HIV were among people ages 20–44, and more than 80% were in the heterosexual population as of 2007 (UNGASS Ecuador, 2008). A 2008 report from the Ministry of Public Health reported that 99% of cases were spread through sexual contact, defining the primary route of transmission of HIV in the country (CARE, 2008). The Pan American Health Organization estimates that 74% of the cases of HIV/AIDS are concentrated in coastal regions (PAHO, 2012). Santa Elena province, the site of the current study, is a coastal province with the third highest HIV incidence in Ecuador at 10.87 cases per 100,000 inhabitants (Ministerio de Salud Pública del Ecuador, 2010). Recent evidence indicates that HIV prevalence in coastal regions of Ecuador may even exceed 1% among pregnant women (Sánchez-Gómez et al., 2013). The available evidence suggests that Ecuador faces a generalized HIV epidemic that is concentrated in coastal regions and predominantly sexually transmitted among the general population (Sánchez-Gómez et al., 2013; UNAIDS, 2008).

The current scope of research on HIV in Ecuador is limited. Prior research has primarily focused on specific populations such as men who have sex with men (MSM) and commercial sex workers (Bautista et al., 2008; Cabezas et al., 2015; Grant et al., 2014; Gutiérrez et al., 2006a; Gutiérrez et al., 2006b; Hernandez et al., 2016; Jacobson et al., 2014; Sánchez-Gómez et al., 2015; Solomon, Smith & del Rio, 2008). While several studies of HIV knowledge, attitudes, and behaviors have been conducted elsewhere in the country, little is known about rural, coastal regions where prevalence rates are among the highest in the country, and general healthcare is less readily available (Cabezas et al., 2013; Chedraui et al., 2007; Dearborn, Lewis & Mino, 2010; PAHO, 2012; Park et al., 2002).

Specifically, research is needed to better understand the potential social and ecological factors influencing the spread of HIV in the general population. Of the models that exist to describe HIV prevention and risk behaviors, several have demonstrated that interpersonal factors, perceived social norms, and social environmental factors substantially influence HIV risk (Ajzen, 1991; Ewart, 1991; Fisher & Fisher, 1992). However, there is also very little known about individual risk perception amongst Ecuadoreans. A deeper understanding of these issues can provide information about factors that might influence the spread of HIV and, in turn, might elucidate the most relevant content for HIV prevention initiatives in the area.

Accordingly, this qualitative study aimed to explore HIV attitudes, risk behaviors, and risk perceptions in the rural, coastal community of Manglaralto, Ecuador. This study was nested within a larger quantitative investigation that broadly sought to understand the Manglaralto community’s knowledge about and behaviors concerning HIV and HIV testing. A sub-set of those who completed the quantitative survey was invited to participate in in-depth interviews to explore these issues further and understand possible individual and contextual factors not easily captured by quantitative surveys. The goal of the current study was to generate insight into actions that could be taken to improve regional HIV prevention, diagnosis, and treatment efforts in this setting.

## Methods

### Setting

The study was conducted from May to August of 2013 at the Manglaralto Hospital, a public hospital in the town of Manglaralto in the Santa Elena province on the Southwestern coast of Ecuador. It is the smaller of two hospitals in the province and serves residents of the northern portion of the province. The Manglaralto Hospital provides anonymous and free HIV testing and counseling to anyone who requests it. This site was selected because the Santa Elena province has the third highest HIV/AIDS prevalence in Ecuador, the hospital is easily accessible by public transportation, and the hospital administration and staff were interested in the issue of HIV in their community. Interviews were conducted at the hospital in a private setting in which participants could openly discuss sensitive issues such as HIV/AIDS and their own possible HIV-associated risk behaviors.

### Study sample

The sample for the qualitative sub-study comprised 15 out-patients (seven men, eight women) who visited the Manglaralto Hospital. They were recruited during medical clinic hours and selected by purposive sampling based on demographic information (gender, age and education) obtained through the larger study’s quantitative survey. Interviewees were not selected in relation to seeking HIV testing or receipt of HIV test results. Rather, it was the goal of this study to assess the customary clinic patients’ understanding about HIV transmission and prevention and their perceived risk of infection. Inclusion criteria for both the main study and this sub-study were that participants be (1) over 18 years old and (2) a resident of the Santa Elena province. Recruitment continued until no additional themes emerged (i.e., saturation).

### Study procedures

Following their completion of the quantitative survey (*n* = 343), purposive sampling was used to select patients for inclusion in this sub-study based on gender, age and education, and invited by a member of the research team to participate in an interview. Approximately 15% of those asked to participate in the interview refused, either for logistical reasons (e.g., did not have time) or unease in being interviewed. All interviews were conducted individually and anonymously. The interviews were audio-recorded and typically lasted 60 min. The interviews were conducted in Spanish by a trained interviewer who was not a native Ecuadorian but had lived in the region for many years and was culturally competent. One other member of the research team (AB, MW, VP or NS), with language proficiency in Spanish, was also present during each interview.

All study participants provided written informed consent prior to the audio-recorded interview. The recordings were subsequently transcribed in Spanish. Any identifying information was removed and replaced with pseudonyms.

In addition to receiving HIV/STI prevention brochures as part of the main study, participants in the qualitative sub-study were also reimbursed for their time in the form of a gift equivalent to US $2 and given a Certificate of Study Completion. The research study was approved by the Human Subjects Committee at Yale University (HSC Protocol #1304011872) and the Board of Directors of the Manglaralto Hospital.

### Interview guide

A draft interview guide, based on existing literature and previous studies in the region, focused on knowledge about HIV, attitudes towards HIV and PLWH, and sexual risk behaviors. Prior to beginning study recruitment, the guide was modified based upon input from local medical staff and patients from Manglaralto Hospital. The guide was further modified to improve cultural sensitivity based on the initial interviews, and additional questions were added to reflect emergent themes.

The domains in the final interview guide were: (1) HIV knowledge; (2) source of HIV knowledge; (3) condom use; (4) HIV risk behaviors; (5) HIV risk perceptions; (6) HIV prevention behaviors; (7) HIV attitudes and stigma; (8) friendships and social interactions with PLWH; and (9) specific suggestions to improve local HIV prevention efforts.

### Analytic methods

Prior to arriving in Ecuador, the research team developed a preliminary coding scheme based on the research questions. During data collection the research team (AB, MW, VP and NS) met daily (and weekly with LG via skype) to review and analyze the data. Codes were added based on emergent themes and existing codes were refined, clarified, or collapsed. This process was done iteratively until the codes were clearly defined and similarly understood by all members of the research team. The first two transcripts were then translated into English, independently coded in ATLAS.ti (version 1.0.2) by two bi-lingual members of the research team (MW, AB) and discussed in meetings with the senior author. Any coding discrepancies were resolved by consensus during these meetings. Once inter-coding reliability was judged to be acceptable, the remaining interviews were then coded in Spanish by MW and AB. A third transcript was translated into English and reviewed (by MW, AB, LG) midway through the coding process to ensure that coders continued to reliably assign codes. Quotes from the transcripts selected for inclusion in the manuscript were translated into English. Given the community’s small size, participants are identified by only an arbitrary participant number, an age range, and gender to protect their anonymity.

Analytical induction and comparative analysis were used to identify common patterns and themes relevant to the research aims (Braun & Clarke, 2006; Clarke et al., 2013). We sought out “negative” instances (i.e., comparative analysis that may not fit initial constructs) in order to expand, adapt, or restrict the original conceptual scheme. Coding and analysis was an iterative process and continued until saturation was reached (i.e., no new themes or information emerged).

## Results

### Study sample

Table 1 provides demographic information about the study sample. The total sample can be characterized as fairly young and minimally educated. The mean age for the total sample was 38.9 years (11.5 SD), although males were approximately five years older than females (41.6 vs. 36.1 years). Half of the total sample had completed only a primary education. Two participants (one male, one female) reported being college educated. Slightly fewer females had secondary education.

**Table 1:** Characteristics of the study sample (*N* = 15).

	Fraction	Percent
Male	7/15	46.7%
Age (years)^a^		
Mean (SD)	N/A	38.9 (11.5)
Education^a^		
Primary	7/14	50%
Secondary	4/14	35.7%
College	2/14	14.3%

**Notes.**

a Data missing for one female participant.

The major themes to emerge from the interview data were perceived negative consequences of being infected with HIV, sexual behaviors that increased the potential for HIV infection, and a tendency to minimize personal risk of infection.

### Negative consequences of being infected with HIV

#### Fear of discrimination and isolation

Interviews revealed a consistent expectation that contracting HIV would lead to isolation and retribution. Participants generally believed that society treats people with HIV poorly. All participants noted the high levels of societal discrimination against PLWH. Participant 1, a male in his 20s, stated that he would not tell other people that he received an HIV test, explaining:

Because you know how society is here… They discriminate against those people [with HIV]… Here in Ecuador the people discriminate a lot against people with those types of sicknesses.

Many participants spoke specifically about their fear of abandonment and discrimination by friends and family if they were to disclose a positive HIV test result. When asked how a positive HIV test result would affect his friendships, Participant 2, a man in his 30s, responded that “My friends, well, one always has friends in the street, but when one most needs them they are no longer friends.” Similarly, Participant 3, a female in her 20s, stated:

[My family would react in] a really bad way. [And friends would react] much worse. Because they would leave you. And, family always protects you and with time maybe they would come to understand… In contrast, friends would always abandon you.

Participants also spoke of their community as being afraid of people with HIV. Participant 4, a male in his 60s, elaborated on the notion of being afraid of people with HIV and consequently leaving them in isolation, explaining:

A person who is infected…. We, or most of us, are afraid to go near them because they can infect you…. And, I have seen in the press, in the movies, that someone who is infected is not treated as important by anyone, so those people are left all alone…. And here it is even worse because no one will help you.

As a result of all the negative associations with HIV, some participants reported that they would want to die if they were diagnosed with HIV. For example, Participant 5, a female in her 30s, noted that suicide was preferable to the abandonment she would face by having the disease.

As exemplified by these quotes, the majority of participants anticipated social isolation and discrimination that would accompany being diagnosed with HIV. The general attitude towards HIV can be summarized as one of perceived suffering and social isolation.

#### HIV as a deadly disease

Almost all participants viewed HIV as an illness that involves much physical suffering and tantamount to receiving a death sentence. As Participant 6, a male in his 40s, noted:

For me…in my case…or for the person that gets detected with the HIV virus…it means to die slowly. I say this because it is a sickness that takes its course. From what I have seen, you fall [ill] and you begin the symptoms bit by bit and, once you realize that, you are already in the terminal stage… I have had some friends with this disease, and I have seen that they are suffering.

### Sexual behaviors that increase risk for HIV infection

#### Multiple sex partners

Many participants, both male and female, described their culture as one in which infidelity is pervasive and the primary way that HIV spreads within the community. Specifically, they identified men as the ones most often engaging in extramarital sex because it was an accepted phenomenon within the culture. No participants stated or suggested that females in this community are unfaithful to their male partners. Typical of others’ opinions, one female participant referred to this practice as “the disobedience of the men”. Other participants elaborated on this theme:

Someone told me that these questions are confidential, so in that case… I have also cheated on my wife, because I am a man. But I don’t want to share more about this. (Participant 6, a male in his 40s.)

It’s not that I have had sex with others… It’s that my husband is a man, and the men sometimes, you know, are going to have…[*Pause*]. Even with trust, one can always walk on another path [*colloquial expression suggesting extramarital sex*] and is going to have sex with someone else, and this person has had sex with another person, and from that you can get infected. (Participant 5, a female in her 30s.)

Infidelity was an acknowledged common occurrence, and participants did not believe that there was anything to be done to counteract it. Interviewees viewed infidelity as a firmly entrenched cultural practice.

#### Lack of condom use

Condom use varied by type of sexual partner. The perception of trust within a relationship often influenced the decision to use or not use condoms. Some men reported using a condom with commercial sex workers or extramarital or casual partners. For example, Participant 1 (male, in his 20s) reported that he would use a condom in the brothels, but that he would not use a condom with his wife since he trusts her.

Many women reported not using condoms with their husbands because of the trust in their marriage and despite infidelity of males being commonly acknowledged. Female participants who believed their husbands were cheating on them hoped that, but were unsure if, their spouses used condoms in their extramarital affairs.

Both male and female participants described a variety of reasons why men prefer not to use condoms. These reasons included issues of decreased sensation and the belief that a female’s desire for a male to use a condom was an indication that she was unfaithful. Participant 7, a woman in her 30s, contextualized the situation for women by saying, “…the truth is, here it is very difficult for you to say to your husband or your boyfriend, ‘Use a condom”’.

#### Perception of personal risk of HIV infection

Many women identified females like themselves as at risk for contracting HIV because of their husbands’ sexual infidelities and the fact that they do not frequently use condoms with their husbands.

I always hear on the television them talking about this sickness [HIV], and I think that, they say that there are thousands of housewives [“*amas de casa”*] who are the most infected because of the men who are unfaithful and are walking around places where they don’t need to be [*expression for extramarital sex*]. So, I think we are the most affected. (Participant 8, a female in her 30s.)

Acknowledging her vulnerability to infection, Participant 9, a female in her 40s, similarly said that women are at increased risk for acquiring HIV because of their husbands’ sexual behavior. She believed she was at risk of contracting HIV because her husband is a fisherman who is often away for periods of 15 days, and she believes he is unfaithful during those absences.

Contrary to women’s risk perceptions, men who stated that they engaged in extramarital sex and did not use protection with their wives still considered themselves to be at low or no risk for infection. They sometimes acknowledged that, in general, people who engaged in these same activities were at risk, but they did not identify themselves as such. One man, Participant 6 (male in his 40s), exemplified this attitude. He had previously identified himself as having sex outside of his marriage and not using a condom with his wife. However, he said he was confident that he was not at risk for HIV although later noting that other men who are unfaithful are at increased risk of contracting HIV. He recognized the theoretical risk associated with having multiple partners but did not recognize his personal risk—even after acknowledging his own infidelity.

## Discussion

This qualitative study assessed HIV attitudes, risk perceptions, and risk behaviors in an open-ended fashion in a rural, coastal region of Ecuador where HIV prevalence is among the highest in the country (Ministerio de Salud Pública del Ecuador, 2010). The data indicated that a positive HIV test result was considered by participants to be a lethal diagnosis, and participants were concerned about potential isolation, discrimination, and erosion of their support system, particularly friendships. Nonetheless, a few participants indicated that family would play an important, positive role in helping PLWH cope. These results regarding community isolation are consistent with other findings in Ecuador and parts of Latin America, which reported isolation and discrimination on the basis of HIV status (Aggleton, Parker & Maluwa, 2003; Ayarza & Reyes, 2002; Manji, Peña & Dubrow, 2007; UNAIDS, 2013). The reported individual- and community-level negative attitudes towards those with HIV are particularly concerning as it may discourage PLWH from disclosing their status to sexual partners and negatively affect their quality of life, as is observed in other parts of the world with high levels of HIV stigmatization (Brooks et al., 2005; Centers for Disease Control and Prevention (CDC), 2000; Chesney & Smith, 1999; Goldin, 1982; Herek, Capitanio & Widaman, 2003; Weiser et al., 2006). If lack of disclosure is accompanied by lack of condom use, the risk of transmission increases, posing a serious public health threat.

Addressing negative attitudes towards those living with HIV may need to be a major part of ongoing HIV testing and educational campaigns in Ecuador. Recommended strategies include implementing educational campaigns that provide explicit information about disease transmission and prevention, reframing HIV/AIDS as a chronic disease with effective treatment options and providing consistent pre- and post-test counseling for HIV screening tests (Mahajan et al., 2008; Obermeyer & Osborn, 2007). It may also be important to fold HIV care and prevention into neutral and confidential healthcare and community settings (Nyblade et al., 2009). Given the negative perceptions about HIV disease, one potential method could be to ensure that HIV services are offered within the framework of general healthcare services, thus allowing patients to request HIV-related services in a discreet and confidential manner. Finally, policy makers might want to further investigate the ways in which HIV-related attitudes in the Santa Elena province and throughout the country are inhibiting HIV testing and prevention efforts. However, additional evidence, particularly quantitative studies, is needed to validate the findings from this qualitative investigation prior to implementing such strategies. Strategies that were developed in other socio-culture contexts may need to be tailored to this new setting.

Another major theme that emerged from the interviews was the high prevalence of risk behaviors. Consistent with reports of *machismo* trends in other parts of Latin America (Nyblade et al., 2009; Sternberg, 2000) participants viewed male infidelity as commonplace and generally accepted in their community. Similar to other studies in Ecuador (Cabezas et al., 2013; Park et al., 2002), some participants in our study minimized their risk of HIV, despite engaging in HIV-associated risk behaviors. Since some men in the community appear to be engaging in sex with multiple partners and having unprotected sex with their spouses, our results support the potential need for condom use campaigns that focus particularly on the importance of men always using condoms with new or casual sex partners. To prevent men from transmitting STIs to their spouses, assertiveness and condom negotiation training may be helpful for women whose husbands are suspected of having multiple partners. Healthcare providers in Ecuador may also benefit from training to consistently screen for risk behaviors in a way that will elicit truthful responses. As of the time of this study, Ecuador was routinely testing only pregnant women for HIV (Ministerio de Salud Pública del Ecuador, 2010). However, the current findings may signal a need for providers to test all patients that report high-risk behaviors. To prevent the HIV epidemic in Ecuador from growing, healthcare providers may need to identify, treat, and counsel patients with HIV. Expanding HIV testing could also reinforce efforts to combat negative attitudes, since increased testing is associated with reduced marginalization (Weiser et al., 2006). It is important to note that additional research is required to validate these findings before designing and implementing interventions specifically for this population.

This study had some potential limitations. First, it is possible that a larger sample size from multiple locations around the region or individuals who specifically sought HIV testing services may have generated further insights into the topics we investigated; however, by the fifteenth interview no new codes or themes were emerging, and therefore no additional interviews were conducted. Second, the study did not specifically target PLWH, and their voice is lacking from the analysis. Instead, this study aimed to understand attitudes within the general population, and future research is needed to understand these issues from the perspective of PLWH as well as for the general population in other coastal regions of Ecuador. Third, since this qualitative study was nested inside a quantitative study, participants could have been influenced by the questionnaire they completed prior to the interview, as it asked questions about HIV attitudes, stigma, and risk. Fourth, this investigation focused on a single population in Manglaralto, Ecuador and cannot be interpreted as indicative of trends across the entire coast or country. Finally, this study was conducted within a hospital setting, so it is not necessarily applicable to those who are not accessing medical care in this community. However, it does reflect the target population for any hospital-based interventions.

This qualitative study examined HIV attitudes, risk perceptions, and risk behaviors in a coastal region of Ecuador. The epidemic can currently be classified as small but at risk of growing. The combination of high levels of negative attitudes towards HIV, prevalence of risky sexual behaviors, and low perception of personal risk identified in this study suggests a dangerous risk profile for the expansion of the HIV epidemic in Ecuador. These findings may suggest a need for increased education about HIV among the general population in Manglaralto, specifically focused on promoting testing, recognizing personal risk and how to reduce that risk, and condom negotiation skills. Efforts in these areas may allow people to better understand the connections between risk behaviors and transmission as well as to counter negative attitudes toward PLWH. Finally, more accurate and updated prevalence estimates by region are needed to more effectively monitor the epidemic in Ecuador. Given the limited data from this region, we recommend epidemiological and social network studies to identify specific at-risk and bridge populations. Studies similar to our own are also needed in other parts of the country to test whether the major themes we identified generalize to other regions and whether HIV prevention interventions developed for the Manglaralto region are relevant to other parts of Ecuador.

## Additional Information and Declarations

### Data Availability

The following information was supplied regarding data availability: As this is a qualitative study, there was no raw data.
